# Applications of artificial intelligence in emergency and critical care diagnostics: a systematic review and meta-analysis

**DOI:** 10.3389/frai.2024.1422551

**Published:** 2024-10-04

**Authors:** Jithin K. Sreedharan, Fred Saleh, Abdullah Alqahtani, Ibrahim Ahmed Albalawi, Gokul Krishna Gopalakrishnan, Hadi Abdullah Alahmed, Basem Ahmed Alsultan, Dhafer Mana Alalharith, Musallam Alnasser, Ayedh Dafer Alahmari, Manjush Karthika

**Affiliations:** ^1^Department of Respiratory Therapy, College of Health Sciences, University of Doha for Science and Technology, Doha, Qatar; ^2^Deanship—College of Health Sciences, University of Doha for Science and Technology, Doha, Qatar; ^3^Department of Respiratory Care, Prince Sultan Military College of Health Sciences, Dammam, Saudi Arabia; ^4^Department of Respiratory Care, Batterjee Medical College, Jeddah, Saudi Arabia; ^5^Department of Respiratory Therapy, Armed Forces Hospital, Dhahran, Saudi Arabia; ^6^Department of Rehabilitation Science, College of Applied Medical Sciences, King Saud University, Riyadh, Saudi Arabia; ^7^Faculty of Medical and Health Sciences, Liwa College, Abu Dhabi, United Arab Emirates

**Keywords:** artificial intelligence, machine learning, critical care medicine, healthcare, diagnosis

## Abstract

**Introduction:**

Artificial intelligence has come to be the highlight in almost all fields of science. It uses various models and algorithms to detect patterns and specific findings to diagnose a disease with utmost accuracy. With the increasing need for accurate and precise diagnosis of disease, employing artificial intelligence models and concepts in healthcare setup can be beneficial.

**Methodology:**

The search engines and databases employed in this study are PubMed, ScienceDirect and Medline. Studies published between 1st January 2013 to 1st February 2023 were included in this analysis. The selected articles were screened preliminarily using the Rayyan web tool, after which investigators screened the selected articles individually. The risk of bias for the selected studies was assessed using QUADAS-2 tool specially designed to test bias among studies related to diagnostic test reviews.

**Results:**

In this review, 17 studies were included from a total of 12,173 studies. These studies were analysed for their sensitivity, accuracy, positive predictive value, specificity and negative predictive value in diagnosing barrette’s neoplasia, cardiac arrest, esophageal adenocarcinoma, sepsis and gastrointestinal stromal tumors. All the studies reported heterogeneity with *p*-value <0.05 at confidence interval 95%.

**Conclusion:**

The existing evidential data suggests that artificial intelligence can be highly helpful in the field of diagnosis providing maximum precision and early detection. This helps to prevent disease progression and also helps to provide treatment at the earliest. Employing artificial intelligence in diagnosis will define the advancement of health care environment and also be beneficial in every aspect concerned with treatment to illnesses.

## Introduction

Artificial intelligence (AI) is increasingly being utilized in healthcare through machine learning algorithms to analyze medical data and improve patient outcomes. AI-powered applications are becoming integral to clinical settings and ongoing research, providing medical professionals with valuable insights and supporting improved health experiences (Beam et al., 2023). The utilization of AI is providing new opportunities for harnessing expanding data sources to improve patient outcomes. AI has the potential to enhance diagnostic precision, predict prognoses more accurately, tailor treatments to specific needs, and optimize the performance of healthcare systems. These applications hold promise for advancing medical care and improving patient experiences (Vollmer et al., 2020). The different types of AI used in healthcare system are machine learning, natural language processing, physical robots, and robotic process automation. The different types of AI used in healthcare system are machine learning, natural language processing (NLP), physical robots, robotic process automation (Rajeev, 2021).

Machine learning (ML) is a type of AI that enables computers to learn from data without explicit programming. By identifying patterns and trends in data, ML algorithms can predict future outcomes and make informed decisions. ML is an essential component of many AI applications, allowing for the creation of predictive models, data classification, and trend identification (Great Learning, n.d.). Data annotation is a crucial element of ML, where it involves labelling data to train algorithms. Natural language processing (NLP) is a branch of AI that enables machines to understand and interpret human language. NLP performs two main functions: processing unstructured data, such as text, and recognizing speech. By using NLP, machines can analyze and learn from human language, making them more effective at completing tasks and improving overall performance (Vollmer et al., 2020). Deep learning is a type of ML that uses layered algorithms to analyze data, providing solutions to problems that traditional MLs cannot solve. It has a broad range of applications in various fields, including NLP, drug development, and disease diagnosis. In healthcare, deep learning algorithms are used to analyze patient medical histories and suggest the most effective therapies, aid in early diagnosis of diseases such as Alzheimer’s, and improve mental health by enabling more accurate disease identification and treatment. With its ability to analyze complex data sets and identify patterns, deep learning is becoming an increasingly valuable tool in healthcare research and practice (Onpassive, 2022).

Medical diagnostics will greatly benefit from such artificially intelligent models. One such example is application of AI in detection of diabetic retinopathy. Early detection of diabetic retinopathy is possible by the AI models due to its ability to analyse images at granular levels which is impossible for ophthalmologists to identify (Basu et al., 2020). Medical imaging relies on the application of machine learning (ML) and deep learning algorithms to accurately predict early symptoms of medical conditions. These algorithms can be used to identify and correct diagnostic or prescription errors by comparing prescriptions to patient health information, thereby improving the accuracy and efficacy of medical treatments (Latif et al., 2019). AI also helps in earlier detection of cancer, early diagnosis of fatal blood disease, treatment for rare disease, automated image diagnosis, participation in clinical trials and diagnosing numerous diseases such as diabetes, chronic heart disease, tuberculosis, stroke, cerebrovascular, cancer, skin diseases and liver diseases (Kumar et al., 2023; Intellipaat, 2023). AI powered virtual assistants are also being utilized in healthcare. It uses ML algorithms and NLP to improve patient experience, supporting healthcare staffs in day-to-day practices and ensure compliance. It can assist patients by answering real-time concerns of their queries regarding the disease and treatment, providing real-time medication reminders and virtual care by booking appointments and aiding in uploading medical documents (Basu et al., 2020).

The application of AI and ML in critical care diagnostics has the potential to revolutionize healthcare. AI and ML algorithms can analyze large volumes of complex patient data in real-time, providing critical care physicians with valuable insights to inform diagnosis and treatment decisions. These algorithms can identify patterns and anomalies in patient data, enabling earlier detection of critical conditions and improving overall patient outcomes. AI-powered imaging technologies, such as CT scans and MRIs, can quickly and accurately identify abnormalities, helping physicians diagnose critical conditions more rapidly (Neoteric, n.d.). Machine learning models can also predict patient deterioration and alert physicians to intervene before a critical event occurs, reducing the likelihood of adverse outcomes. Additionally, AI and ML can facilitate remote monitoring of critical care patients, allowing physicians to track vital signs and other health indicators from a distance and intervene as necessary. Overall, the integration of AI and ML into critical care diagnostics has the potential to significantly improve patient outcomes, enhance diagnostic accuracy, and optimize treatment strategies (Linkedin, n.d.). Despite the potential benefits of AI and ML in critical care diagnosis and management, there are still significant gaps in knowledge and research surrounding their use in the intensive care unit (ICU) setting. Some of the key challenges include the need for robust and diverse data sets to train algorithms, the potential for bias in algorithm development, and the need to integrate AI and ML technologies into existing clinical workflows. Additionally, the ethical implications of using these technologies in critical care, such as issues related to data privacy and informed consent, require careful consideration.

Our study aims to review the current literature and provide a comprehensive analysis of the effectiveness, safety, and feasibility of AI-based technologies in ICU diagnosis. By examining the existing evidence base, the study will provide insights into the potential clinical impact of AI-based technologies and identify opportunities for further research and development in this critical area of healthcare.

## Methodology

### Study search strategy

This review was conducted adhering to preferred reporting items for systematic review and meta-analysis (PRISMA) guidelines (PROSPERO Reg. ID: CRD42023422745), which is an evidence-based set of recommendations aimed to ensure systematic reviews are conducted and reported with accuracy, allowing for a clear understanding of the review’s rationale, methodology, and findings (Moher et al., 2009). This study employed a comprehensive and systematic search strategy, utilizing well-established search engines and databases such as PubMed, ScienceDirect, and Medline. The search was focused on identifying relevant literature pertaining to the use of AI in critical care medicine, with a specific emphasis on evaluating the accuracy and benefits of AI-based technologies in critical care diagnostics. Through a rigorous and exhaustive search process, this study aimed to identify all relevant articles and data sources related to the use of AI in critical care diagnostics, and to synthesize this information in a manner that would enable a comprehensive evaluation of the current state of knowledge in this field, studies published between 1st January 2013 to 1st February 2023 were included in this analysis. The key terms used in this strategized search include, “artificial intelligence and critical care medicine,” “artificial intelligence and healthcare,” “artificial intelligence and diagnosis,” and “machine learning and diagnosis.” Other terms used in place of AI are “digital image analysis,” “computer aided algorithm,” “neuron network” and “deep learning.” These terms were used in combination with “AND” and “OR.” The selected articles were screened preliminarily using the Rayyan web tool, after which investigators screened the selected articles individually (Ouzzani et al., 2016).

### Inclusion and exclusion criteria

The studies included in this systematic review and meta-analysis were selected based on a pre-defined set of criteria. First, only studies involving participants above 18 years of age (considered as adult subjects) were included. Second, studies involving the use of AI in the diagnosis of ailments were considered eligible. Third, studies showing rates of true positive, false positive, true negative, and false negative were included. Fourth, studies with histopathological assessment as a standard reference were selected. Fifth, study type was limited to either retrospective or prospective. Sixth, only studies that showed results of the chosen computer-aided algorithm or other AI model were included. Finally, for meta-analysis, studies that derived data from the performed analysis were chosen. By adhering to these rigorous criteria, this review aimed to identify high-quality studies that provided reliable and valid evidence on the use of AI in critical care diagnostics and management. In order to ensure that the studies included in this study are of high quality and relevance, certain exclusion criteria were applied. First, studies with no inference or data derived from their meta-analysis were excluded. Second, studies including subjects with concomitant disease conditions were excluded to ensure that the data analyzed were specific to the ailment being studied. Third, studies with incomplete data were excluded as they may yield unreliable or incomplete results. Fourth, studies with an improper randomization process were excluded to ensure that the data analysed were of sufficient quality to provide meaningful insights. Finally, studies that were not peer-reviewed, such as conference abstracts, white papers, or unpublished theses, studies published in languages other than English, studies that were considered outdated or had been superseded by more recent, comprehensive research were excluded to ensure the quality and reliability of the included research.

### Data extraction

**Figure 1:**
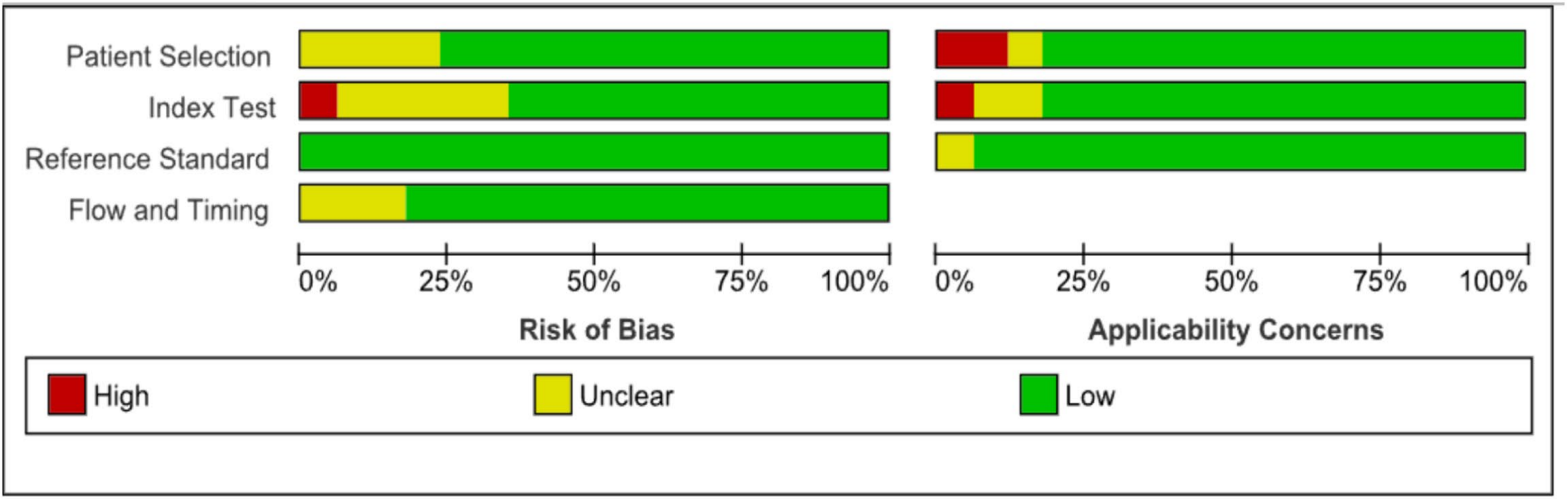
Assessment of quality of selected studies using QUADAS-2 (graph plot).

**Figure 2:**
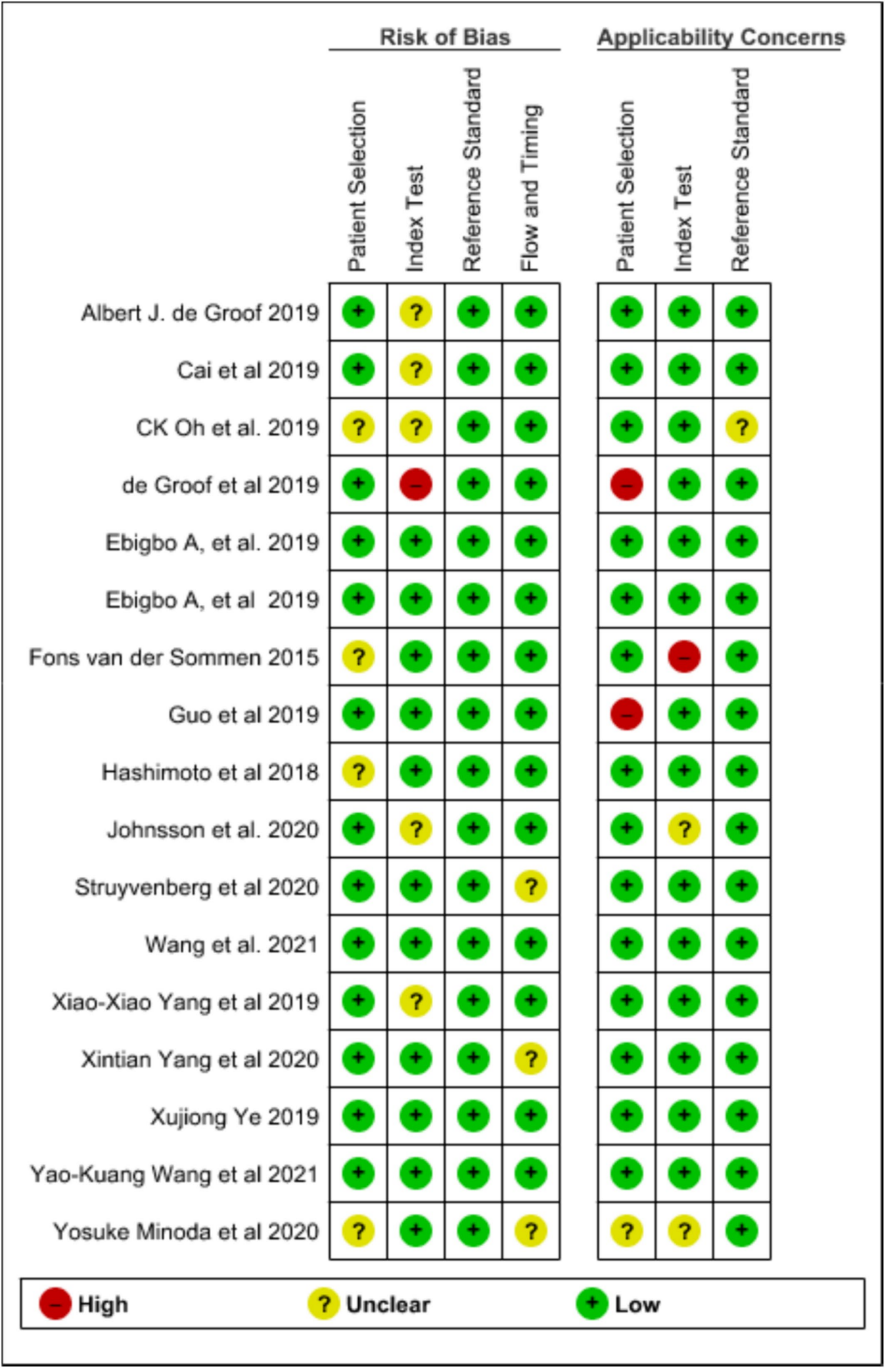
Assessment of quality of selected studies using QUADAS-2 (summary plot).

The investigators screened all the selected studies using Microsoft EndNote X9 to organize and manage references, ensuring an efficient and systematic approach to handling citations and Rayyan online software for systematic reviews, facilitated the blinded and independent screening of titles and abstracts by three independent reviewers, enhancing the rigor and transparency of our selection process. With the help of these tools, the selected articles were either chosen for this review or excluded. The basic characteristics of the studies like year of the study, type of AI model employed, standard reference used, disease diagnosed, type of study conducted and site of the study were collected. The quality of the selected studies was assessed using Quality for Assessment of Diagnostic Studies (QUADAS) scoring tool (Whiting Penny et al., 2011). This tool is specifically designed to evaluate the quality of primary diagnostic accuracy studies, complementing rather than replacing the data extraction process. It should be used alongside the extraction of primary data, such as study design and results, for comprehensive analysis in the review. The tool covers four key domains: patient selection, the index test, the reference standard, and the flow and timing of patients through the study, including the timing of the index tests and reference standard. The domains chosen for assessment in this review include, quality of selection of patients, quality of flow, quality of reference standard and quality of timing. The results of the domains assessed were categorised as low, unclear or high risk. Figure 1 represents graph plot of the risk of bias assessed using QUADAS-2 tool while Figure 2 shows the summary plot of the same. For data extraction, three independent reviewers were involved in extracting the data from the included studies. Any conflicts or discrepancies between the reviewers during data extraction were resolved through discussion. If the reviewers were unable to reach a consensus, a fourth reviewer was consulted to make the final decision. This process helped us to mitigate biases and ensure the integrity of the extracted data.

**Figure 3:**
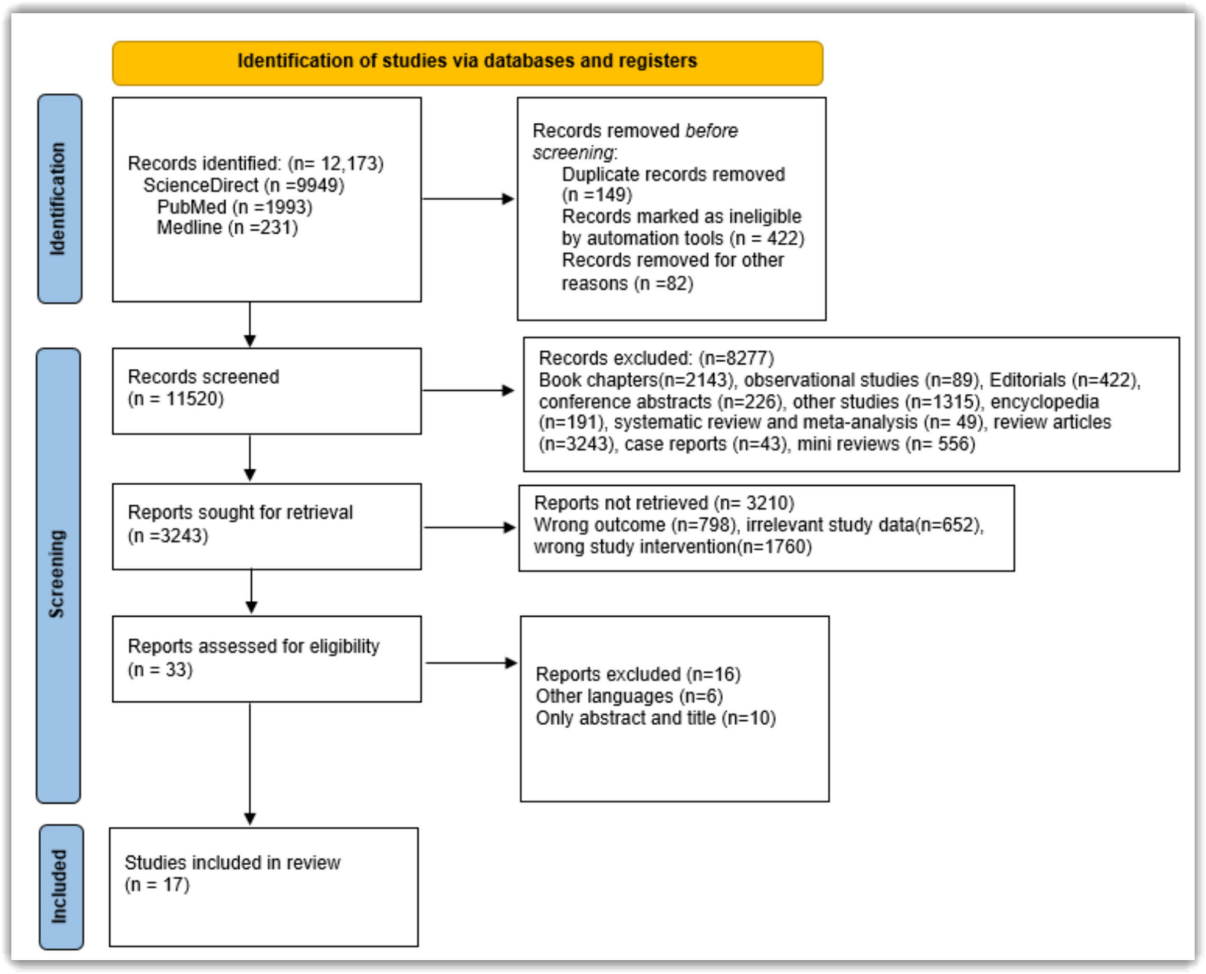
PRISMA flowchart depicting the flow of selection of studies.

Figure 3 illustrates the study selection process conducted in accordance with PRISMA guidelines.

### Study outcomes

The review focused on evaluating the diagnostic accuracy of AI algorithms in critical care settings. Key outcomes assessed included positive likelihood ratio (PLR), negative likelihood ratio (NLR), sensitivity, specificity, and area under the curve (AUC) or summary receiver operating characteristic (SROC) curve. These metrics help determine the effectiveness and reliability of AI models in detecting and ruling out critical conditions. Likelihood ratios provide insight into the impact of test results on disease probability, making them valuable for assessing AI models in critical care diagnostics.

### Statistical analysis

In this meta-analysis, we calculated pooled values of specificity, sensitivity, positive likelihood ratio, negative likelihood ratio, and summary receiver operating characteristic curve (SROC) of the AI algorithms employed in the chosen studies using a random effects model. The random effects model accounts for variability among studies by assuming that the effects being estimated in different studies are not identical but follow a distribution. This approach provides more generalized results, especially when there is heterogeneity among study outcomes. To address heterogeneity, we conducted subgroup analyses based on key study characteristics (e.g., population demographics, intervention types) and performed sensitivity analyses by systematically excluding studies with outlier results. These methods allowed us to explore potential sources of heterogeneity and assess the robustness of our findings. RevMan 5.4 (The Cochrane Collaboration, 2020, London, United Kingdom) statistical software was utilized for all statistical evaluations in this study. RevMan 5.4 is a comprehensive software tool designed for the preparation and maintenance of systematic reviews and meta-analyses, providing functionalities for data synthesis and analysis.

Heterogeneity between the studies was assessed based on several factors identified through subgroup analysis, including the number of participants and studies, type of AI used, year of publishing, and imaging techniques. Heterogeneity was further assessed using regression analysis according to the guidelines issued by Cochrane. The evaluations were made with a 95% confidence interval to ensure the reliability of the results. We used the chi-square test to analyse the heterogeneity of the studies. The chi-square test is a statistical method used to determine if there is a significant association between categorical variables, helping to identify whether variations in study outcomes are due to chance or other factors. By incorporating these tools and methods, we ensured a rigorous and comprehensive statistical analysis of the AI algorithms’ diagnostic performance in the included studies.

## Results

The search strategy yielded 12,173 records from PubMed, ScienceDirect and Medline. After sequential and systematic search and filtration, 17 studies in total were included for this review. Although only 17 studies were included, it is important to note that the methodology followed strict inclusion criteria and systematic review guidelines, to ensure the validity of the objectives. A detailed flow of study selection is depicted in Figure 3. The 17 studies included in this review, focused on the accuracy and ability of AI to detect and diagnose various disease conditions like Barrette’s neoplasia, gastrointestinal stromal tumour, cardiac arrest, esophageal adenocarcinoma and sepsis. Table 1 shows the characteristic features of all the studies chosen for this review. we define the types of studies included in our analysis for clarity. Prospective studies involve following participants over time, starting with the exposure, and observing them into the future to assess outcomes, aiming to establish cause-and-effect relationships in real-time settings. Conversely, retrospective studies examine past data or medical records to explore associations between exposures or treatments and outcomes that have already occurred. Pilot studies, on the other hand, are small-scale preliminary investigations conducted to assess the feasibility, time, cost, and effectiveness of larger studies, typically refining research methods before larger-scale implementation. Finally, the term “standard field” refers to established practices, methodologies, or protocols widely accepted within specific fields of study or research areas, providing a foundational context for our review and analysis. The outcomes assessed from the chosen studies are shown in Table 2 and are explained in detail. Table 3 presents the results of meta-regression analyses assessing heterogeneity across studies. Statistical significance was determined at *p* = 0.05 with a 95% confidence interval for most variables, indicating significant heterogeneity among studies based on factors such as study characteristics, type of AI used, year of publication, and imaging techniques. Notably, the analysis of the number of participants did not reach statistical significance (*p* > 0.05), suggesting that this factor did not significantly contribute to heterogeneity in our study sample. These findings are crucial for understanding the variability in diagnostic accuracy observed across different studies included in our meta-analysis (Table 3).

**Table 1:** Characteristic details of the studies included.

S. No.	Study	Year of study	Type of study	Country	Condition diagnosed	Standard	AI model
1	de Groof et al. (2019)	2019	Prospective	Netherlands	Barrett’s Neoplasia	Histopathology	Deep learning algorithm
2	van der Sommen et al. (2016)	2015	Pilot	Netherlands	Barrette’s neoplasia	Histopathology	ANN
3	de Groof et al. (2020)	2019	Prospective	Netherlands	Barrett’s neoplasia	Histopathology	Deep learning algorithm
4	Struyvenberg et al. (2021)	2020	Retrospective	Netherlands	Barrett’s neoplasia	Histopathology	Deep learning algorithm
5	Ghatwary et al. (2019)	2019	Retrospective	UK	Early esophageal adenocarcinoma	Histopathology	CNN
6	Ebigbo et al. (2019)	2019	Retrospective	Germany	Esophageal adenocarcinoma	Histopathology	CNN
7	Xia et al. (2021)	2018	Pilot study	USA	Barrett’s neoplasia	Histopathology	CNN
8	Ebigbo et al. (2020)	2019	Retrospective	Germany	Barrett’s neoplasia	Histopathology	CNN
9	Oh et al. (2021)	2019	Retrospective	Korea	Gastrointestinal stromal tumors and leiomyoma	Histopathology	CNN
10	Yang et al. (2022)	2020	Retrospective	China	Gastrointestinal stromal tumors and leiomyoma	Histopathology	Deep learning algorithm
11	Wang D. et al. (2021)	2021	Retrospective	China	Sepsis	Laboratory tests	Random forest algorithm
12	Minoda et al. (2020)	2020	Retrospective	Japan	Gastrointestinal stromal tumors	Histopathology	CNN
13	Wang Y. K. et al. (2021)	2021	Pilot study	Taiwan	Barrett’s neoplasia	Histopathology	CNN
14	Cai et al. (2019)	2019	Retrospective	China	Esophageal adenocarcinoma	Histopathology	DNN
15	Okagawa et al. (2022)	2019	Retrospective	China	Esophageal adenocarcinoma	Histopathology	CNN
16	Guo et al. (2020)	2019	Prospective	Multicentred (China, USA, India)	Esophageal adenocarcinoma	Histopathology	Deep learning algorithm
17	Johnsson et al. (2020)	2020	Prospective	Sweden	Cardiac arrest	ECG	ANN

**Table 2:** Outcomes assessed from selected studies.

S. No.	Study	Year of study	Condition diagnosed	Sensitivity	Specificity	Positive predictive value (PPV)	Negative predictive value (NPV)
1	de Groof et al. (2019)	2019	Barrett’s neoplasia	95%	85%	NA^a^	NA
2	van der Sommen et al. (2016)	2015	Barrette’s neoplasia	86%	87%	50%	50%
3	de Groof et al. (2020)	2019	Barrett’s neoplasia	91%	89%	NA	NA
4	Struyvenberg et al. (2021)	2020	Barrett’s neoplasia	88%	78%	NA	NA
5	Ghatwary et al. (2019)	2019	Early esophageal adenocarcinoma	73.2%	78.2%	NA	NA
6	Ebigbo et al. (2019)	2019	Esophageal adenocarcinoma	97%	88%	NA	NA
7	Xia et al. (2021)	2018	Barrett’s neoplasia	90%	80%	89.2%	98%
8	Ebigbo et al. (2020)	2019	Barrett’s neoplasia	83.7%	100%	NA	NA
9	Oh et al. (2021)	2019	Gastrointestinal stromal tumors	100%	85.7%	95.2%	100%
10	Yang et al. (2022)	2020	Gastrointestinal stromal tumors	98%	94.4%	94.2%	98.1%
11	Wang D. et al. (2021)	2021	Sepsis	87%	89%	NA	NA
12	Minoda et al. (2020)	2020	Gastrointestinal stromal tumors	91.7%	100%	84.6%	72.1%
13	Wang Y. K. et al. (2021)	2021	Barrett’s neoplasia	96.2%	70.4%	92.7%	82.6%
14	Cai et al. (2019)	2019	Esophageal adenocarcinoma	97.8%	85.4%	86.4%	97.6%
15	Okagawa et al. (2022)	2019	Esophageal adenocarcinoma	97.4%	99.4%	92.5%	98%
16	Guo et al. (2020)	2019	Esophageal adenocarcinoma	98%	95%	40%	62%
17	Johnsson et al. (2020)	2020	Cardiac arrest	98%	97%	NA	NA

a NA, not applicable as missing details from the papers.

**Table 3:** Statistical analysis of subgroups.

Parameter	*p*-value
Number of participants	0.02
Number of studies	0.0003
AI model used	0.005
Year of publishing	0.00001
Techniques of imaging	0.0007

### AI in diagnosis of Barrette’s neoplasia

**Figure 4:**
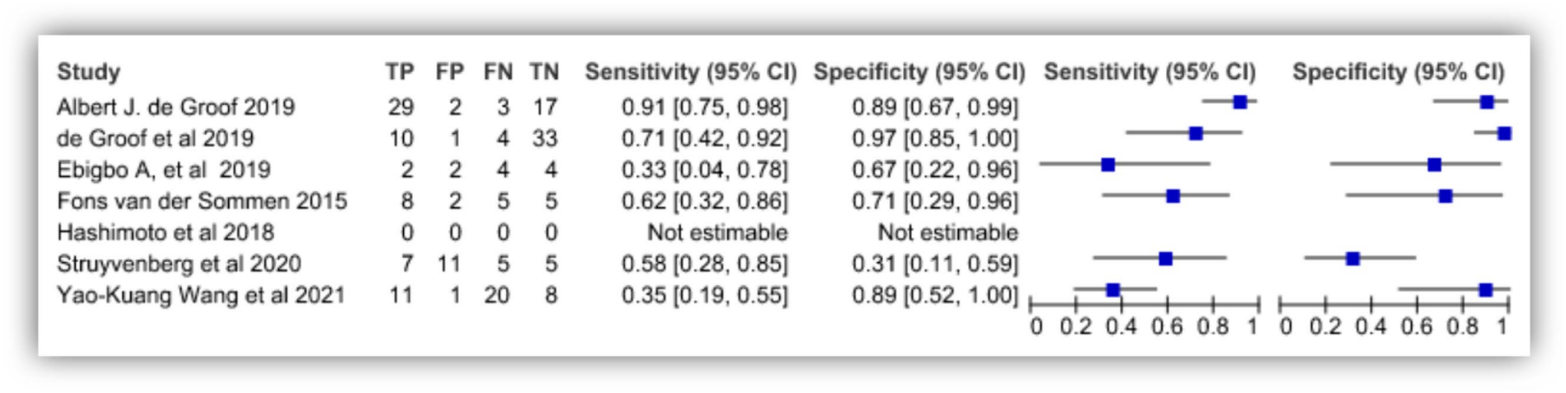
Accuracy of AI diagnosis in Barrette’s neoplasia (forest plot).

**Figure 5:**
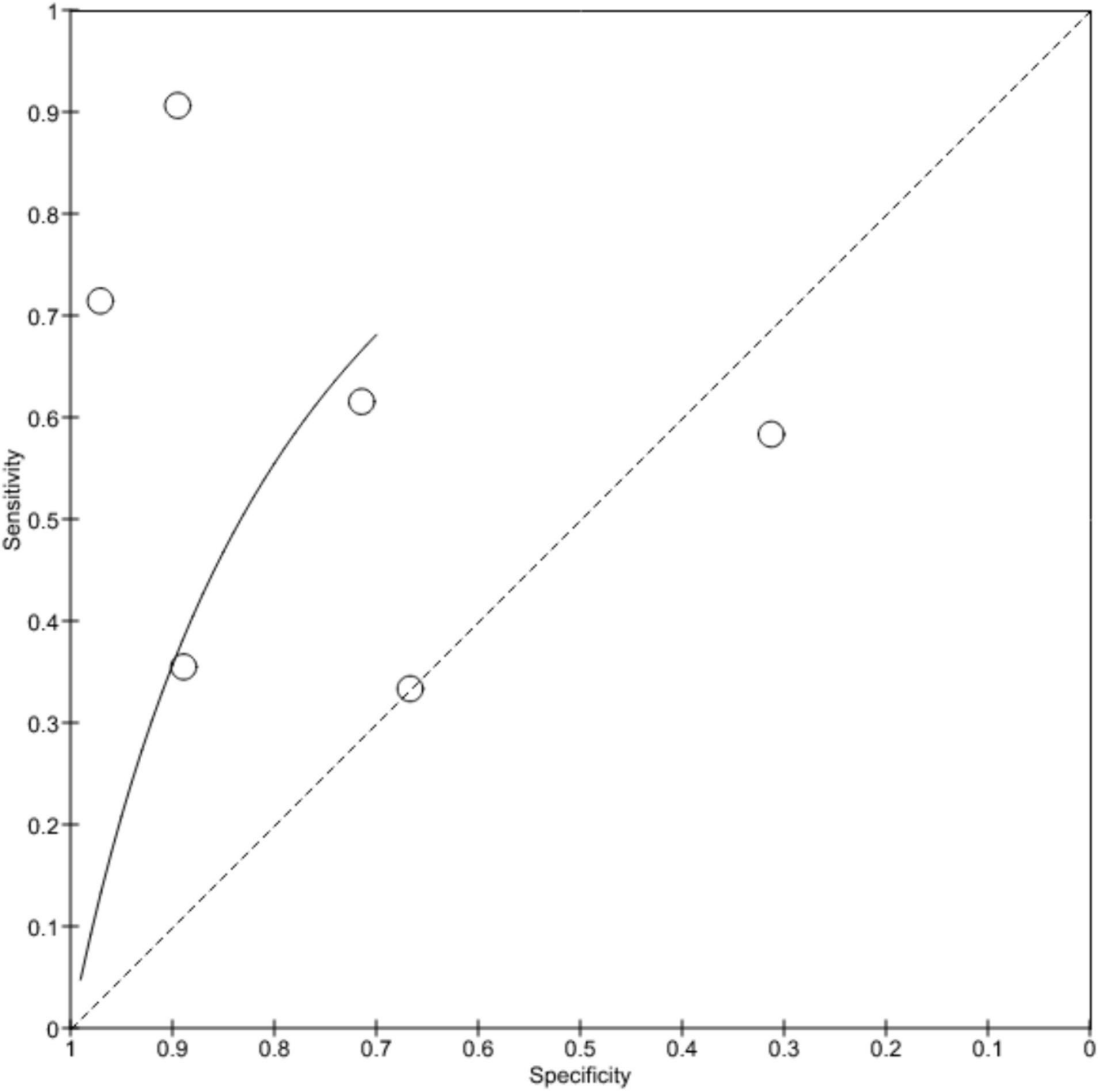
Accuracy of AI diagnosis in Barrette’s neoplasia (SROC plot).

The review included seven studies that analyzed the use of AI for diagnosing Barrett’s neoplasia. All of these studies utilized histopathology as the reference standard Among these studies, de Groof et al. (2019, 2020) conducted two studies and Struyvenberg et al. (2021) conducted one study that used a deep learning algorithm to aid in the diagnosis of Barrett’s neoplasia. Another study by van der Sommen et al. (2016) employed an artificial neural network (ANN) model to diagnose Barrett’s neoplasia. Studies conducted by Xia et al. (2021), Ebigbo et al. (2020), and Wang Y. K. et al. (2021) used convolutional neural network (CNN) model in diagnosis. de Groof et al. (2019) reported 95% sensitivity and 85% specificity while providing no data on the positive predictive value or positive likelihood ratio and negative predictive value or negative likelihood ratio. van der Sommen et al. (2016) reported 86% sensitivity, 87% specificity, 50% positive predictive value and 50% negative predictive value.

de Groof et al. (2020) showed 91% sensitivity and 89% specificity with no predictive values and likelihood ratios. Study by Xia et al. (2021) was observed with 90% sensitivity and 80% specificity with 89.2% positive predictive value and 98% negative predictive value. Ebigbo et al. (2020) reported 83.7% sensitivity and 100% specificity in the diagnosis while Wang Y. K. et al. (2021) showed 96.2% sensitivity, 70.4% specificity with 92.7% positive predictive value and 82.6% negative predictive value. Struyvenberg et al. (2021) shows 88% sensitivity and 78% specificity. Figure 4 depicts the true positive, false positive, true negative and false negative values observed from each study and details a forest plot of results derived from the studies via meta-analysis. Figure 5 illustrates SROC plot of the accuracy of AI to detect Barrette’s neoplasia.

### AI in diagnosis of gastrointestinal stromal tumor

**Figure 6:**
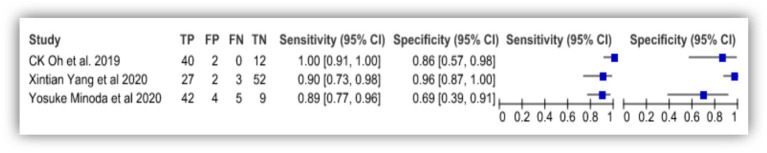
Accuracy of AI diagnosis in gastrointestinal stromal tumor (forest plot).

**Figure 7:**
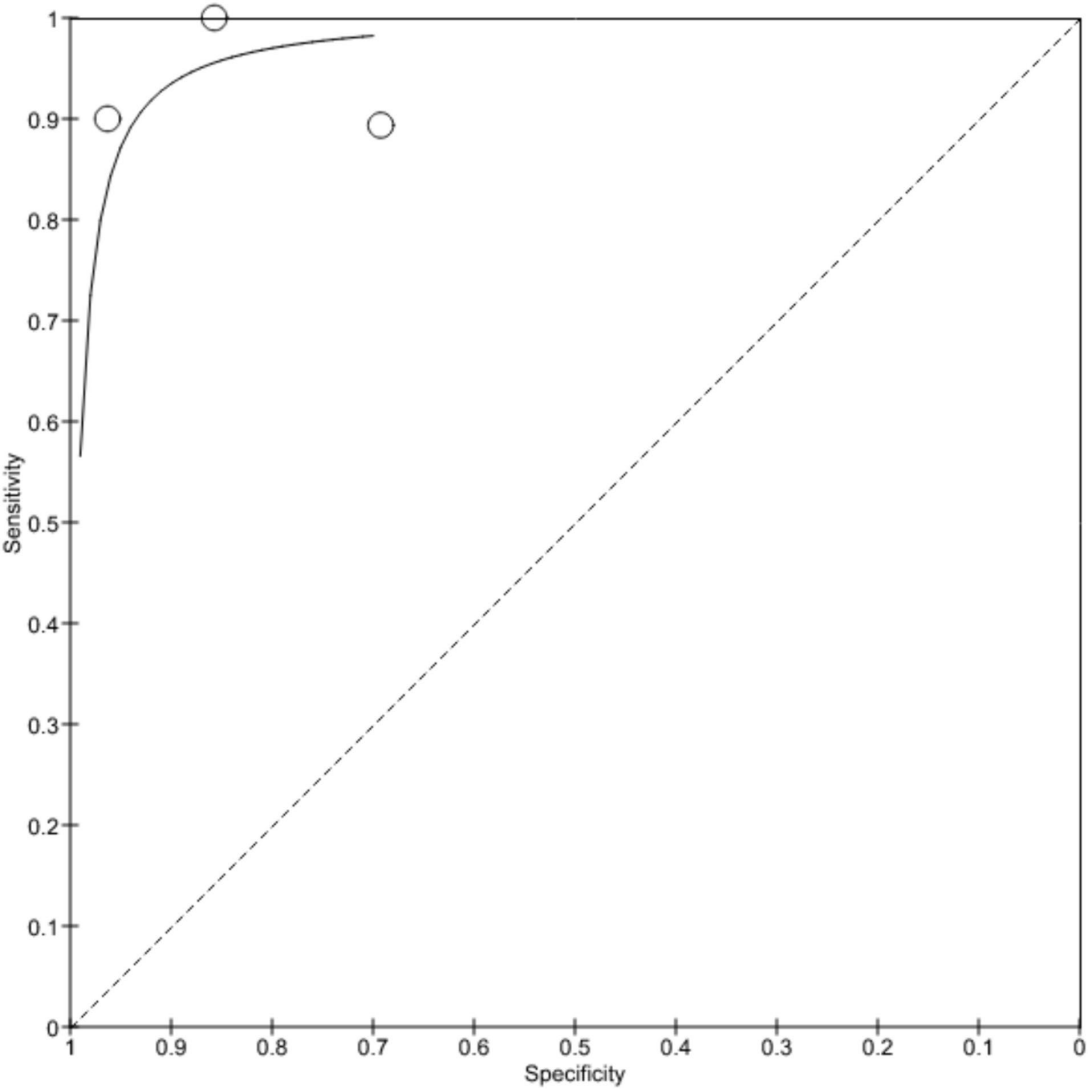
Accuracy of AI diagnosis in gastrointestinal stromal tumor (SROC plot).

Three studies in this review studied the efficacy of AI to detect gastrointestinal stromal tumor (GIST). All three studies used histopathological results as their standard reference for comparison. Oh et al. (2021) and Minoda et al. (2020) developed CNN model for diagnosis of GIST while Yang et al. (2022) used deep learning algorithm to diagnose the condition. The study by Oh et al. (2021) reported 100% sensitivity and 85.7% specificity with 95.2% positive predictive value and 100% negative predictive value. While the study conducted by Yang et al. (2022) showed 98% sensitivity, 94.4% specificity, 94.2% positive predictive value and 98.1% negative predictive value. The study by Minoda et al. (2020) showed 91.7% sensitivity, 100% specificity, 84.6% positive predictive value and 72.1% negative predictive value. Figure 6 shows the forest plot of AI accuracy in diagnosis of GIST and Figure 7 shows the SROC plot of the same.

### AI in the diagnosis of esophageal adenocarcinoma

Five studies selected for this review involved analysing the accuracy of AI in detecting esophageal adenocarcinoma. All the studies used histopathological finding as their standard reference for comparison. Studies by Ghatwary et al. (2019), Ebigbo et al. (2019), and Okagawa et al. (2022) used ANN model to detect adenocarcinoma while a study by Cai et al. (2019) employed deep neural network (DNN) and study by Guo et al. (2020) used deep learning algorithm to detect esophageal adenocarcinoma.

**Figure 8:**
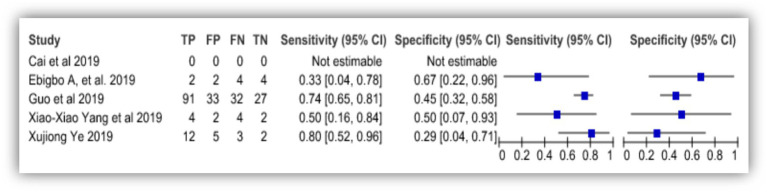
Accuracy of AI diagnosis in esophageal adenocarcinoma (forest plot).

**Figure 9:**
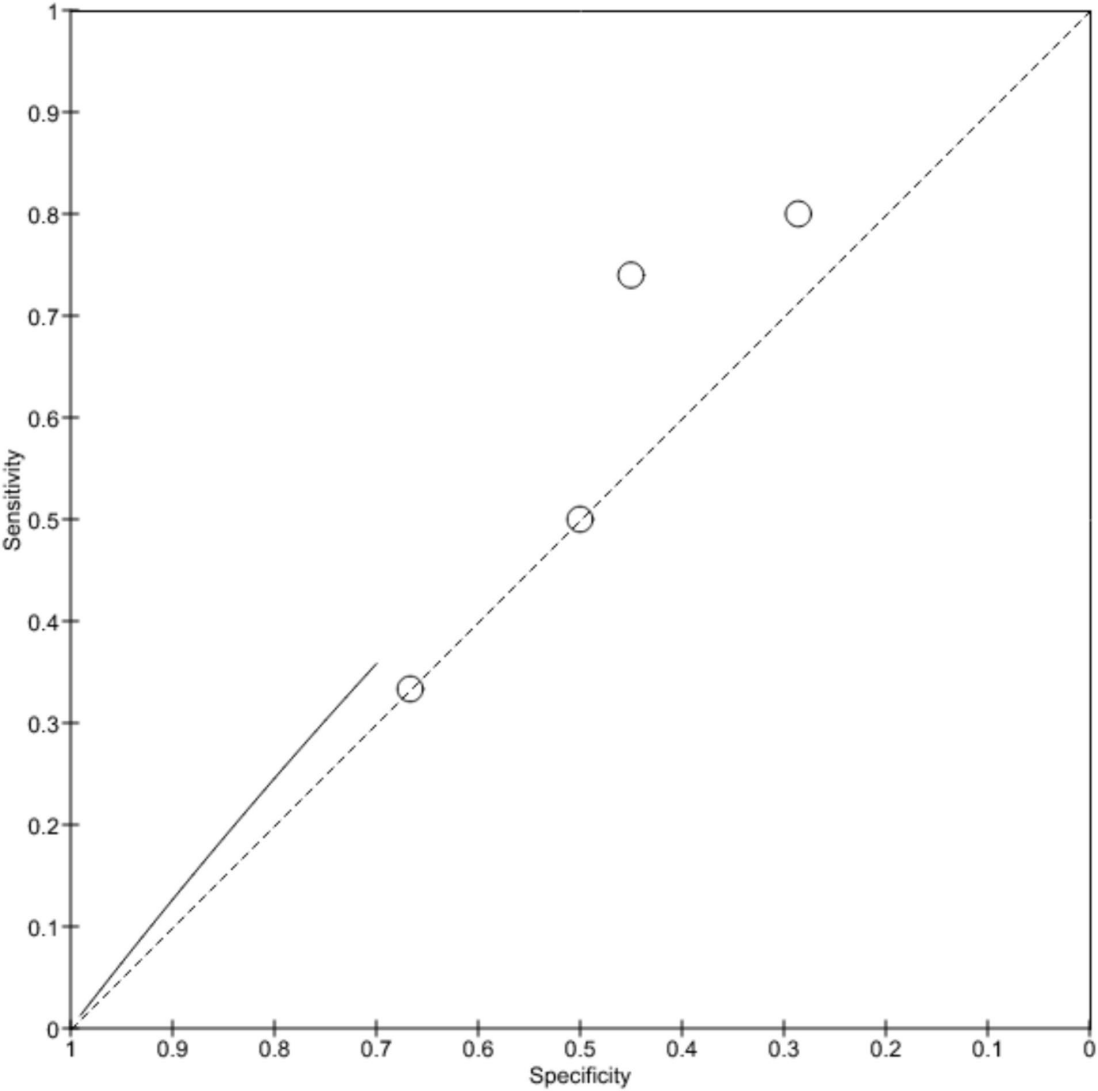
Accuracy of AI diagnosis in esophageal adenocarcinoma (SROC plot).

The study conducted by Ghatwary et al. (2019) reported 73.2% sensitivity and 78.2% specificity of ANN model in detection of adenocarcinoma. Study by Ebigbo et al. (2019) showed 97% sensitivity and 88% specificity. Cai et al. (2019) concluded that DNN model showed 97.8% sensitivity, 85.4% specificity, 86.4% positive predictive value and 97.6% negative predictive value. Okagawa et al. (2022) reported that the AI model employed showed 97.4% sensitivity, 99.4% specificity, 92.5% positive predictive value and 98% negative predictive value. Another study conducted by Guo et al. (2020) reported that the model showed 98% sensitivity, 95% specificity, 40% positive predictive value and 62% negative predictive value. Figure 8 depicts the forest plot showing accuracy of AI diagnosing esophageal adenocarcinoma and Figure 9 shows the SROC plot of the same.

### AI in diagnosis of sepsis

Although, only one study conducted by Wang D. et al. (2021) was found to have been reported and discussed the extent of detection of sepsis by AI algorithm. The AI model employed in the diagnosis was a developed random forest algorithm and the routine standard laboratory tests were used as reference. This study reported a sensitivity of 87% and specificity of 89% in the diagnosis of sepsis by the developed model. The study however provides no data on the positive and negative predictive values or likelihood ratio which aid to analyse the extent of detection of positive and negative cases.

### AI in diagnosis of cardiac arrest

Similar to sepsis, only one study was reported with AI being employed in the detection of heart related complications. Here the study by Johnsson et al. (2020) employed ANN model to diagnose cardiac arrest in participants. The study also used ECG as the reference standard to compare their results. The study reported that the ANN model used for diagnosis showed 98% sensitivity and 97% specificity giving no data on predictive values and likelihood ratios.

## Discussion

AI has emerged as a promising tool in healthcare, particularly in the field of diagnosis. Advanced AI-based techniques and models have been developed to provide high-definition results and accurate diagnosis. Numerous studies have been conducted to evaluate the accuracy of diagnosis using AI and to predict its efficacy in comparison to conventional diagnostic methods. These studies have demonstrated that AI can provide highly accurate and precise diagnoses, thereby enhancing the quality of healthcare outcomes. Incorporating AI into healthcare can not only improve the accuracy of diagnosis but also facilitate the development of personalized treatment plans, resulting in better therapeutic outcomes. The use of AI in healthcare has the potential to revolutionize the field of medicine and significantly improve patient care.

Currently, a considerable number of studies are being conducted to investigate the efficacy of AI in the detection of tumors. Early and accurate diagnosis of tumors using AI can not only be economically beneficial but also aid in preventing the progression of the disease to advanced stages. However, the majority of studies conducted thus far have been limited to the detection of tumors in the gastrointestinal system. Through a critical analysis of the existing literature, this review seeks to demonstrate the potential of AI in enhancing the accuracy and precision of diagnosis, thereby improving patient outcomes. The findings from this review suggest that the integration of AI into clinical practice has the potential to revolutionize the field of medicine and significantly improve patient care.

In the studies included for assessing the extent of accuracy provided by AI in diagnosing Barrette’s neoplasia, almost all the studies yielded good results and was significantly correct on comparison with the traditional diagnostic methods. Conventionally, for the detection of barrette’s neoplasia, a biopsy from the susceptible area of esophagus is collected and then histopathological studies are conducted to detect changes in the tissues and to identify neoplasia. Studies reported that the specific AI models used in their respective studies show sensitivity over 80% and specificity over 70%. Some studies lacked the data on positive and negative predictive values which can help us to identify the extent to which the specific developed models can detect positive and negative cases. Four out of seven selected studies reported no data on predictive values or positive and negative likelihood ratios. Three studies had data on predictive values and showed over 80% positive and negative predictive values. These results demonstrate that the detection of Barrette’s neoplasia using AI developed models provided earlier and better diagnosis of the condition when compared to conventional methods (Visaggi et al., 2022).

In this review we included studies to assess the AI models employed in the diagnosis of sepsis and cardiac arrest. With only limited studies published on both conditions, the results from these studies provide positive hope on AI models to be used in diagnosis of the same. Though the quantified evidence may seem insufficient, the results superior in quality and suggest that AI can be used in diagnosis and also insist that diagnosis by AI is accurate with sensitivity and specificity reported over 80% in both the studies.

The studies chosen to understand the efficiency of AI in diagnosing esophageal adenocarcinoma were observed to show that the models used for the diagnosis were good in accuracy, sensitivity and specificity. These results were compared to the traditional diagnostic methods and were found to be a little more accurate than the later. The selected studies used ANN, DNN, and deep learning algorithm models to diagnose adenocarcinoma and these models showed over 85% specificity and over 85% sensitivity in diagnosis except for one study which showed 73% sensitivity and 78% specificity, however they provided data on positive predictive value and negative predictive value which can aid to detect the true positive, true negative, false positive and false negative cases reported by the AI models.

Preservation and Incorporation of Valuable Endoscopic Innovations (PIVI) initiated by American Society of Gastrointestinal Endoscopy requires the diagnostic methods to either be equally effective or improve the quality of testing. The AI models used in the studies qualify the requirements posed by PIVI and are hence considered to be better choice of diagnostic options (Sharma et al., 2012).

Three studies were included in this review to assess the quality and efficiency of AI models to diagnose gastrointestinal stromal tumor. CNN and deep learning algorithm models were developed and used in diagnosing GIST. The studies over 90% sensitivity and over 85% specificity. These studies also showed 84–95% positive predictive value and 72–100% negative predictive value. The results gathered from these studies are supportive of AI being incorporated in critical care medicine to aid in diagnosis due to its benefits in terms of maximum accuracy and precision, early detection, sensitivity and specificity (Liu et al., 2022).

Considering the promising outcomes demonstrated by the application of AI in critical care diagnostics, several key areas for future research and development warrant attention. Current research predominantly focuses on conditions such as Barrett’s neoplasia, sepsis, cardiac arrest, esophageal adenocarcinoma, and gastrointestinal stromal tumors. Future studies should aim to encompass a broader spectrum of diseases, thereby demonstrating the utility and versatility of AI diagnostic models across diverse medical conditions and healthcare settings. Longitudinal research is essential to evaluate the long-term effects of AI diagnostic tools on patient outcomes, healthcare costs, and system efficiency. These studies should investigate not only immediate diagnostic accuracy but also the long-term benefits and challenges associated with integrating AI into routine clinical practice. There is a critical need for standardizing and externally validating AI models. This includes the development of standardized protocols for training and testing AI models and validating these models across various clinical environments and patient populations to ensure their reliability and generalizability. Further studies should explore the practical aspects of incorporating AI tools into clinical workflows. This involves understanding barriers to adoption, such as the compatibility of AI with existing healthcare systems, user training requirements, and the establishment of regulatory and ethical guidelines to ensure the safe and effective use of AI in diagnostics. There is potential for developing comprehensive AI platforms that combine diagnostic capabilities with other clinical decision support tools. Such platforms could integrate patient data from multiple sources, including electronic health records, imaging, and laboratory results, to provide a holistic view of patient health and support informed clinical decisions. Economic evaluations of AI diagnostic tools are crucial. Future studies should compare AI-driven diagnostics with conventional methods, considering factors such as implementation costs, potential savings from early diagnosis, and improved patient outcomes. By addressing these areas, the research community can work towards realizing the transformative potential of AI in critical care diagnostics, ultimately leading to enhanced patient care and outcomes.

### Major limitations

This study has several limitations including the number of studies analysed due to the inclusion criteria. First, it primarily focuses on specific conditions such as Barrett’s neoplasia, sepsis, cardiac arrest, esophageal adenocarcinoma, and gastrointestinal stromal tumours, which limits the generalizability of findings across a broader spectrum of diseases. Second, there is a lack of long-term studies to evaluate the enduring effects of AI diagnostic tools on patient outcomes, healthcare costs, and overall system efficiency, hindering a comprehensive understanding of AI’s long-term benefits and challenges. Additionally, the study highlights variability in the performance of different AI models, with some studies lacking data on positive and negative predictive values, complicating the assessment of true diagnostic accuracy and reliability. Lastly, there is a critical need for standardized protocols and external validation of AI models across diverse clinical settings and patient populations to ensure their widespread applicability and effectiveness in real-world scenarios.

## Conclusion

In conclusion, the use of AI for diagnosis in healthcare has shown great potential in providing accurate and precise diagnoses, particularly in critical care units. The specific design of AI models for each condition being diagnosed is crucial for their efficacy. Despite the limited number of studies conducted on the use of AI in critical care medicine and diagnostics, the results suggest that AI models can provide outstanding precision, accuracy, sensitivity, and specificity in diagnosing various disease conditions. Several real-world applications underscore this potential. For instance, AI algorithms have been successfully implemented in detecting sepsis in critical care settings, leading to earlier interventions and improved patient outcomes. Similarly, AI-based diagnostic tools for detecting pneumonia from chest X-rays have shown comparable accuracy to human radiologists, aiding in faster and more reliable diagnoses in busy clinical environments.

However, the clinical application of AI is not without challenges. Issues such as data privacy, ethical concerns, and algorithmic bias must be carefully addressed to ensure that AI tools are both effective and equitable. Moreover, the integration of AI into healthcare systems requires rigorous validation, regulatory approval, and continuous monitoring to prevent unintended consequences. While early detection and accurate diagnosis with AI can help prevent disease progression and aid in providing treatment at the earliest possible stage, the successful implementation of AI in healthcare will depend on addressing these challenges. As AI technology continues to advance, further studies are needed not only to fully realize its potential but also to mitigate its limitations. Developing AI models that meet the specific diagnostic requirements of various medical conditions in critical care management, while also ensuring fairness and protecting patient rights, will be essential for the future of AI in healthcare.

## Data Availability

The original contributions presented in the study are included in the article/supplementary material, further inquiries can be directed to the corresponding author.
